# A Precise Nanostructure of Folate-Overhung Mitoxantrone DNA Tetrahedron for Targeted Capture Leukemia

**DOI:** 10.3390/nano10050951

**Published:** 2020-05-16

**Authors:** Ying-Zi Bu, Jia-Rui Xu, Qian Luo, Ming Chen, Li-Min Mu, Wan-Liang Lu

**Affiliations:** State Key Laboratory of Natural and Biomimetic Drugs, Beijing Key Laboratory of Molecular Pharmaceutics and New Drug System, School of Pharmaceutical Sciences, Peking University, Beijing 100191, China; eiko@pku.edu.cn (Y.-Z.B.); xujr@bjmu.edu.cn (J.-R.X.); lq-ql@pku.edu.cn (Q.L.); cm_cally@pku.edu.cn (M.C.); liminmu@bjmu.edu.cn (L.-M.M.)

**Keywords:** folate-overhung DNA tetrahedron, mitoxantrone, cellular uptake, co-localization of nuclei, leukemia

## Abstract

Regular chemotherapy cannot eliminate leukemic cells, due to the sparse distribution of cancer cells in leukemia patients. Here, we report a precise nanostructure of folate-overhung mitoxantrone DNA tetrahedron that enables the treatment of leukemic cells by targeted action. Folate is used as a targeting molecule and synthesized with DNA strand in forming the folate-overhang DNA complement, and the complement is then separately base-paired onto six sides of the fabricated DNA tetrahedron. Mitoxantrone is used as an anticancer agent and intercalated into the double strands of the folate-overhung DNA tetrahedron for drug loading. The evaluation studies are performed on leukemia BALL-1 and K562 cells. The results demonstrate that the folate-overhung mitoxantrone DNA tetrahedra (approximately 25nm) are able to target leukemic cells, transport across the nuclei membrane, induce the apoptosis, and enhance the overall efficacy of treating leukemic cells in vitro and in leukemia-bearing mice. This study provides a potential drug-containing DNA nanostructure, to clean the sparsely distributed leukemic cells in patients.

## 1. Introduction

Based on Watson–Crick base paring, it is possible to generate immobile junctions or even three-dimensional DNA, rather than the linear duplexes on common occasions [1]. DNA nanotechnology exhibits a promising prospect, because of its precise fabrication, stability, high biocompatibility and low immunogenicity [2]. It is proposed as an engineering material and a carrier of genetic information in living cells. Several types of DNA nano-assemblies have been fabricated, including tile-based structures, DNA origami [3], DNA tetrahedron [4], DNA octahedron [5], DNA cube [6], molecular machines, and DNA computers [7] for trying to solve the issues in the fields of nano-medicines, X-ray crystallography, nuclear magnetic resonance spectroscopy, etc. However, DNA-based conception and technical innovations still need further clarification.

Leukemia is a malignant cancer with high mortality. Because of the sparse distribution of leukemic cells in the patients, current comprehensive treatments, including surgical, chemo-/radio, and bone marrow transplantation therapy, cannot eliminate leukemic cells. Among the comprehensive treatments, chemotherapy plays a crucial role to sweep leukemic cells. However, regular chemotherapy cannot eliminate the leukemic cells, due to the absence of specific capture capability.

In this study, we hypothesized that the folate-overhung DNA tetrahedra could be used for targeted cleaning of the leukemic cells. In this nanostructure, mitoxantrone was used as the anticancer drug, and folate was used as the targeting molecule, by conjugating DNA overhang compliment strands to improve cellular uptake of drug. Further, the folate-overhung DNA tetrahedra were also designed to improve the co-localization of drug with nuclei, because mitoxantrone mainly took effect in the nuclei of cancer cells. Meanwhile, this drug-containing DNA nanostructure was proposed to induce cancer cell apoptosis for potentiating the efficacy in treating leukemic cells.

Mitoxantrone is an anthracycline drug used in treating various cancers, such as solid tumors, acute leukemia, lymphoma, prostate and breast cancer [8]. Mitoxantrone is a type II topoisomerase inhibitor, and it disrupts DNA synthesis and DNA repair in both healthy cells and cancer cells by intercalation between DNA bases [9,10]. Because of extensive bio-distribution after drug administration, mitoxantrone exhibits a strong systemic toxicity, especially a severe cardiotoxicity [11]. Accordingly, the application of mitoxantrone is limited by its adverse reaction in the treatment of cancer.

Folate, also named vitamin B9, cannot be synthesized inside mammalian animal cells, and mainly relies on the intake of extrinsic sources. It is reported that folate is involved in DNA synthesis and repair [12], and is able to lower the risk of cardiovascular disease and to reduce newborn congenital heart defects during pregnancy [13]. Folate can bind specifically with folate receptor, which is known as the high affinity membrane folate-binding protein as well. The overexpression of folate receptor has been evidenced in membranes of cancers, including those of ovarian cancer, breast cancer, sarcomas and acute myeloid leukemia [14]. Consequently, folate is used as a specific ligand for the targeted chemotherapy of cancer. This is because there exists a high affinity between folate receptor and folate. Besides, this high affinity is not affected by the conjugation of folate with other molecules, such as folate complexes.

In the present study, the objectives were to synthesize a folate-overhung DNA tetrahedron through base-pairing a newly synthesized folate-DNA overhang complement derivative, and to indicate the effects and mechanism in the treatment of leukemia.

## 2. Materials and Methods

### 2.1. Materials and Cell Lines

Sulfosuccinimidyl 4-(N-maleimidomethyl) cyclo hexane-1-carclohexane-1-carboxylate (sulfo-SMCC) and mitoxantrone were purchased from Harveybio, Co. Ltd. (Beijing, China). N-succinimidyl 3-(2-pyridyldithio) propionate (SPDP) was purchased from Merck-Millipore (Beijing local agent, China). Tris (2-carboxyethyl) phosphine hydrochloride was purchased from Adamas (Beijing local agent, China). N-ethylmaleimide was purchased from Sigma-Aldrich (Beijing local agent, China). All oligonucleotides and cell counting kit-8 (CCK-8) were supplied by Sangon Biotech (Shanghai, China). Caspase-3 and caspase-8 colorimetric assay kits were purchased from Applygen Technologies Inc. (Beijing, China). Dimethyl sulphoxide (DMSO) was purchased from Sigma-Aldrich (Beijing local agent, China). Fetal bovine serum (FBS) was purchased from Life Technologies, Co., Ltd. (Nanjing, China). Other reagents were commercially available, and used without additional purification.

Leukemia BALL-1 and K562 cells were kindly provided by Professor Guo-Rui Ruan of Hematology Institute at Peking University People’s Hospital. The cells were cultured in RPMI 1640 medium, containing 10% FBS, under a humidified atmosphere of 5% CO_2_ at 37 °C.

### 2.2. Conjugation of Folate to DNA Overhang Complement

Folate was conjugated to the amino-modified overhang complement strand, as described by Erben et al. [15] Sulfo-SMCC and SPDP were used in the synthesis process as heterobifunctional cross-linkers. Amino-modified oligos overhang complement was re-suspended in phosphate buffered saline (137 mM NaCl 2.7 mM KCl, 8 mM Na_2_HPO_4_ and 2 mM K_2_HPO_4_, PBS pH 7.4), at a final concentration of 1mM. The DNA solution was combined with a saturated sulfo-SMCC solution (2.9 mg/mL) at 1:2 (*v*/*v*) in PBS (pH 7.4), and incubated for 1 h. Bio-Rad Micro Bio-Spin P-6 columns were used to remove excess cross-linkers. Folate was dissolved in PBS (pH 7.4) to 1 mM, combined with SPDP solution (5 mg/mL in DMSO) at 25:2 (*v*/*v*) and incubated for 1 h. Tris(2-carboxyethyl) phosphine hydrochloride was dissolved in PBS (pH 7.4) and added to folate solution at 1:10 (*v*/*v*). After incubation for 30 min, DNA and folate solutions were combined and incubated at 4 °C overnight. N-ethylmaleimide was dissolved in PBS (pH 7.4) and added to DNA-folate conjugate solution at 1:25 (*v*/*v*), to bind any unreacted thiol groups. After incubation for 30 min, a Bio-Rad Micro Bio-Spin P-6 column was used to transfer the conjugates into TM buffer (10 mM Tris-HCl and 5 mM MgCl_2_, pH 8). The product was confirmed by using MALDI-TOF-MS (Shimadzu, Japan).

### 2.3. Synthesis and Characterization of DNA Tetrahedra

Blank DNA tetrahedra, blank folate-overhung DNA tetrahedra, mitoxantrone DNA tetrahedra, and folate-overhung mitoxantrone DNA tetrahedra were prepared as the following.

To prepare blank DNA tetrahedra, equal amounts of stand 1−6 were combined to a final concentration (1 mM) of each oligonucleotide in the TM buffer. Annealing was performed by holding the mixture at 60 °C for 3 min, and followed by cooling to 4 °C for approximately 30 s.

To prepare blank folate-overhung DNA tetrahedra, equal amounts of stand 1–6 with overhangs at the 3’ end with 6 folds of overhang complements were combined to a final concentration of 1 mM. The following procedures were the same as those of the blank tetrahedra.

To prepare mitoxantrone DNA tetrahedra, blank DNA tetrahedra were constructed first and cooled to 4 °C overnight. An adequate amount of mitoxantrone was added to blank DNA tetrahedra at 1:1 (*v*/*v*) and incubated at room temperature for 6 h. Mitoxantrone DNA tetrahedra were then obtained. For epirubicin DNA tetrahedra used for indicating cellular uptake and the co-localization of nuclei, the procedures were the same as above, except mitoxantrone was replaced with epirubicin.

To simulate the structure of DNA tetrahedra, the software uniquimer 3D was used based on energy minimization (Department of Chemical Engineering, Hong Kong University of Science and Technology, Hong Kong, China).

To prepare folate-overhung mitoxantrone DNA tetrahedra, the procedures were similar to those of mitoxantrone DNA tetrahedra, except for replacing blank DNA tetrahedra with blank folate-overhung DNA tetrahedra. The same folate-overhung epirubicin DNA tetrahedra were prepared for indicating the cellular uptake and co-localization of nuclei.

The morphology of mitoxantrone DNA tetrahedra, folate-overhung DNA tetrahedra and folate-overhung mitoxantrone DNA tetrahedra were further observed under a transmission electron microscope (TEM, FEI Company, Hillsboro, OR, USA).

The encapsulation efficiency (EE) was measured by the following procedures. Different formulations were centrifuged at 10,000 revolutions per minute (rpm) for 10 min. Mitoxantrone concentration in the supernatant was measured by the UV-visible spectrophotometer. The EE was calculated using the following formula: EE (%) = (Wtotal − Wsup)/Wtotal × 100%, where Wsup is the amount of mitoxantrone in the supernatant, and Wtotal is the total amount of mitoxantrone added in the formulation.

In vitro leaky rate of mitoxantrone from mitoxantrone DNA tetrahedra or from folate-overhung mitoxantrone DNA tetrahedra was performed by dialysis against the release medium (PBS, pH 7.4), at 37 °C. Mitoxantrone content in the release medium was measured by spectrophotometer at 610 nm. The leaky rate (%) was calculated using the formula: leaky rate = (Wi/Wtotal) × 100%, where Wi is the measured amount of mitoxantrone at the 24 h in the release medium and Wtotal is the total amount of mitoxantrone in the equal volume of DNA tetrahedra prior to dialysis. Each assay was repeated in triplicate.

The stability of folate-overhang mitoxantrone DNA tetrahedra was characterized by the following procedures. Folate-overhang mitoxantrone DNA tetrahedra was dissolved in serum protein-containing medium (PBS buffer containing 10% fetal bovine serum). It was incubated at 37 °C in cell culture incubator for 0 h, 1 h, 2 h, 3 h, 5 h, 7 h and 23 h. PAGE experiments were performed to verify stability.

### 2.4. Polyacrylamide Gel Electrophoresis

Tris(hydroxymethyl)aminomethane (Tris)-boric acid-ethylene diamine tetraacetic Na4 (TBE, 10×) buffer was made by adding double distilled H_2_O in 108 g Tris base, 55 g boric acid and 9.3 g EDTA-Na4 to 1 liter (final pH 8.3). Native gels were run in 1 × TBE buffer. A volume (12.5 ml) of acrylamide (8%) was made and vortexed, consisting of 3.3 ml 30% acrylamide: bisacrylamide solution (29:1, *v*/*v*), 1.25 ml 10 × TBE buffer, and 7.9 mL H_2_O. Then, a volume of 7 μL tetramethylethylenediamine (TEMED) and a volume of 63 μL 10% ammonium persulfate (APS) were added into the above solution, vortexed, and used as the polyacrylamide gel for electrophoresis.

The DNA samples were mixed with the loading buffer and then loaded in the wells of gels in the electrophoresis apparatus. The gel was run at 80 V until the bromophenol blue dye had migrated two-thirds the length of the gel. Then, the gel was stained and photographed.

### 2.5. Cellular Uptake by Leukemic Cells

Leukemia BALL-1 and K562 cells were seeded in 6-well plates at a cell density of 7000/well. Folate (100 μM) was pre-added in some wells. The cells were incubated under 5% CO_2_ at 37 °C for 24 h. Epirubicin DNA tetrahedra, and folate-overhung epirubicin DNA tetrahedra were added into separate wells and incubated under 5% CO_2_ at 37 °C for 4 h. The same amount of folate-overhung epirubicin DNA tetrahedra was added in the wells, and folate was added beforehand. The final concentration of epirubicin was 10 μM, and PBS (pH 7.4) was used as the blank control. After incubation, the cells were collected and re-suspended in PBS (pH 7.4). The fluorescence intensity was measured by the FACScan flow cytometer (Becton Dickinson Biosciences, Franklin Lakes, NJ, USA). Excitation wavelength was set at 488 nm and emission wavelength was set at 560 nm.

### 2.6. Transport Across Nuclei Membrane of Leukemic Cells

Leukemia BALL-1 and K562 cells were seeded in 24-well plates at a cell density of 106/well. The cells were incubated under 5% CO_2_ at 37 °C for 24 h. Epirubicin DNA tetrahedra and folate-overhung epirubicin DNA tetrahedra were added into separate wells and incubated under 5% CO_2_ at 37 °C for 2 h. The final concentration of epirubicin was 20 μM, and PBS (pH 7.4) was used as the blank control. After incubation, the cells were collected and washed three times by using cold PBS (pH 7.4). The cells were incubated with Hoechst 33258 (2 μg/mL) at room temperature for 1 h, by avoiding light. And then the cells were washed twice using PBS (pH 7.4). Re-suspend the cells in 200 μL PBS in the confocal wells. The cells were observed by using the confocal laser fluorescent microscope with Leica confocal software (TCS-SP8, LASAF 3.1, Wetzlar, Germany).

### 2.7. Cytotoxicity to Leukemic Cells

To compare cytotoxicities of varying formulations including DNA tetrahedra, mitoxantrone DNA tetrahedra, and folate-overhung mitoxantrone DNA tetrahedra, leukemia BALL-1 and K562 cells were seeded in 96-well culture plates at a density of 104/well for BALL-1 cells, and a density of 7000/well for K562 cells. The cells were cultured under 5% CO_2_ at 37 °C for 24 h, and subsequently treated with DNA tetrahedra and serial concentrations of drug formulations. The final concentration of mitoxantrone was in the range of 0–5 μM in the formulations. PBS (pH 7.4) was used as the blank control. After treatment for 48 h, the inhibitory effects were determined by using CCK-8 assay. Survival rates were calculated using the following formula: survival (%) = (A450 nm for treated cells/A450 nm for blank control cells) × 100%, where A450 nm is the absorbance at 450 nm, as measured by a microplate reader.

### 2.8. Induction of Apoptosis in Leukemic Cells

Apoptosis was detected using a fluorescein isothiocyanate annexin V staining kit (KeyGen Biotechnology Co. Ltd., Nanjing, China). Briefly, leukemia BALL-1 and K562 cells were seeded into 6-well culture plates at 10^6^/well and cultured under 5% CO_2_ at 37 °C for 24 h. The cells were treated with free mitoxantrone, DNA tetrahedra, mitoxantrone DNA tetrahedra and folate-overhung mitoxantrone DNA tetrahedra for 10 h. The final concentration of mitoxantrone was 10 μM. Control experiments were done by adding PBS. After incubation, the cells were stained using Annexin V-Fluor 647 and 7AAD, then assessed according to manufacturer instructions by the FACScan flow cytometer (Becton Dickinson Biosciences, Franklin Lakes, NJ, USA). Each assay was repeated in triplicate.

### 2.9. Apoptotic Signaling Pathway Induced in Leukemic Cells

To study the apoptotic signaling pathway in leukemic cells induced by drug treatment, caspase-8 and caspase-3 activities were measured. Briefly, leukemia BALL-1 cells and K562 cells were seeded into 24-well culture plates at 106/well and cultured under 5% CO_2_ at 37 °C. After incubation for 24 h, the cells were treated with mitoxantrone DNA tetrahedra and folate-overhung mitoxantrone DNA tetrahedra, and further incubated for 12 h. The final concentration of mitoxantrone was 10 μM. Control experiments were done by adding PBS (pH7.4). Then, cells were processed according to the manufacturer instructions of caspase colorimetric assay kits (KeyGen Biotechnology Co. Ltd., Nanjing, China). Each assay was repeated in triplicate.

### 2.10. Anticancer Efficacy in Mice

Male BALB/c nude mice (20 g above) were included for the studies. All of the animal experiments adhered to the principles of care and use of laboratory animals and were approved by the Institutional Animal Care and Use Committee of Peking University (SYXK 2016-0041). Briefly, approximately 107 leukemia BALL-1 cells were re-suspended in 200 μL physiological saline, and injected subcutaneously into right flank of mouse. At day 9, the tumor masses were observed clearly and the tumor-bearing mice were randomly divided into five groups (6 per group). At days 9, 11, 13, 15, 17 and 19 post-inoculation, free mitoxantrone, mitoxantrone DNA tetrahedra, and folate-overhung mitoxantrone DNA tetrahedra were administered to mice by peritumoral injection. The dose for each formulation was 1 mg mitoxantrone / kg mouse body weight. Physiological saline was administered as a blank control. Tumor volumes were measured every day with a caliper. Tumor volumes were evaluated with the formula: V = length × width^2^ × 0.5 (mm^3^). The weights of the mice were measured every day from day 1 to day 20. At day 16, the blood indicators were examined.

### 2.11. Statistical Analysis

Data were expressed as the mean ± standard deviation (SD). Analysis of variance (ANOVA) was used to determine the significance among groups, among which post hoc tests with the Bonferroni correction were used for multiple comparisons. A value of *p* < 0.05 or < 0.1 was considered as statistically significant.

## 3. Results

### 3.1. Fabricating Folate-Overhung Mitoxantrone DNA Tetrohedron

**Figure 1:**
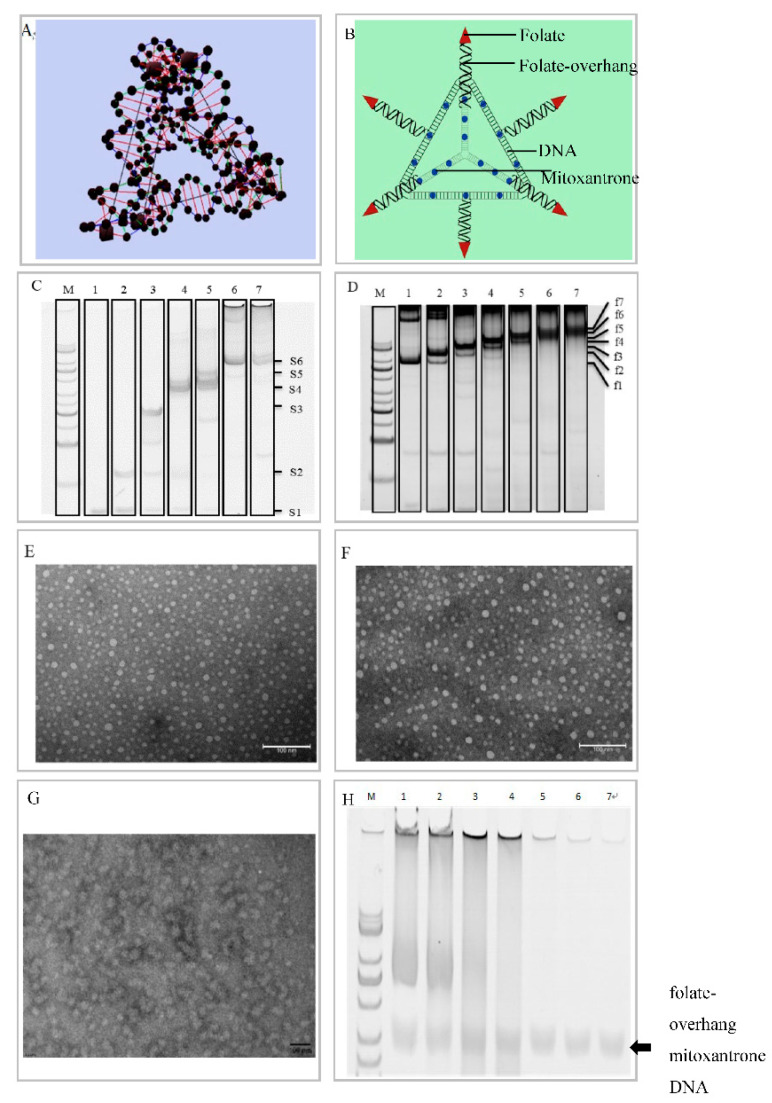
Precise synthesis of folate-overhung DNA tetrahedra. (**A**). The simulated image of DNA tetrahedra; (**B**). The schematic representation of folate-overhung DNA tetrahedra; (**C**). Polyacrylamide gel electrophoresis of the DNA tetrahedra formation; lane 1, strand 1 (S1); lane 2, strand 1-2 (S1-2); lane 3, strand 1-3 (S1-3); lane 4, strand 1-4 (S1-4); lane 5, stand 1-5 (S1-5); lane 6 and lane 7, stand 1-6 (S1-6). For lane 1-6, the DNA strands were base paired under the PCR condition; for lane 7, the DNA strands were base paired at room temperature. Lane M is the marker lane. (**D**). Lane 1, 0 folate per DNA tetrahedron (f1); lane 2, 1 folate per DNA tetrahedron (f2); lane 3, 2 folates per DNA tetrahedron (f3); lane 4, 3 folates per DNA tetrahedron (f4); lane 5, 4 folates per DNA tetrahedron (f5); lane 6, 5 folates per DNA tetrahedron (f6); lane 7, 6 folates per DNA tetrahedron (f7). Lane M is the marker lane. (**E**). The TEM image of the mitoxantrone DNA tetrahedra; (**F**). The TEM image of the folate-overhung DNA tetrahedra; (**G**). The TEM image of the folate-overhung mitoxantrone DNA tetrahedra; (**H**). The stability of folate-overhung mitoxantrone DNA tetrahedra within 24h. The folate-overhung mitoxantrone DNA tetrahedra was dissolved in PBS containing 10% FBS and incubated at 37 °C for different periods of time. Lane 1,0 h; lane 2,1 h; lane 3, 2 h; lane 4, 3 h; lane 5, 5 h; lane 6, 7 h; lane 7, 23 h; lane M, marker.

We firstly synthesized the DNA tetrahedra, and further, the folate-overhung DNA tetrahedra, by assembling 6 oligonucleotides by the polymerase chain reaction (PCR). To assemble the folate-overhung DNA tetrahedra, folate was conjugated to the DNA overhang complement strand first (Table S1). The MALDI-TOF-MS spectrum demonstrated that the difference of molecular weight between the product folate-overhang complement (Figure S1) and the overhang complement was equal to that of folate (MW = 441), thus indicating a successful synthesis. Then, 6 oligonucleotides with overhangs were mixed with the folate-overhang complements, and the folate-overhung DNA tetrahedra were then formed. Two kinds of DNA tetrahedra structures are illustrated by the simulated and schematic representations (Figure 1A,B). The native polyacrylamide gel electrophoresis (PAGE) analysis showed the step-wise assembly of DNA tetrahedra as each strand was added (Figure 1C). The DNA tetrahedron can bind precisely with 6 folate molecules (Figure 1D). By varying oligonucleotides with overhangs or not, the DNA tetrahedra were formed in the range of 0–6 folate molecules per tetrahedron. In the study, we mainly focused on the folate-overhung DNA tetrahedra, which had 6 folate molecules per tetrahedron. The transmission electron microscope (TEM) images of mitoxantrone DNA tetrahedra (Figure 1E), folate-overhung DNA tetrahedra (Figure 1F) and folate-overhung mitoxantrone DNA tetrahedra (Figure 1G) showed that these tetrahedra were successfully assembled with approximately 25 nm in size. The encapsulation efficiency of mitoxantrone in two kinds of mitoxantrone-containing DNA tetrahedra was above 85%, and the content of mitoxantrone in two kinds of mitoxantrone-containing DNA tetrahedra was around 1.0 mg/mL, respectively. The leaky rate of mitoxantrone from the DNA tetrahedra was below 10% (Table S2). Folate-overhang mitoxantrone DNA tetrahedra was dissolved in PBS containing 10% FBS and incubated at 37 °C for different periods of time. (Lane 1, 0 h; lane 2, 1 h; lane 3, 2 h; lane 4, 3 h; lane 5, 5 h; lane 6, 7 h; lane 7, 23 h; lane M, marker.) PAGE experiments were performed to verify the stability, and FBS (10%)-contained PBS buffer was selected as a medium to simulate the blood environment in animals. The results showed that the folate-overhang mitoxantrone DNA tetrahedra were stable under the simulated environment (Figure 1H).

### 3.2. Targeted Uptake by Leukemic Cells

**Figure 2:**
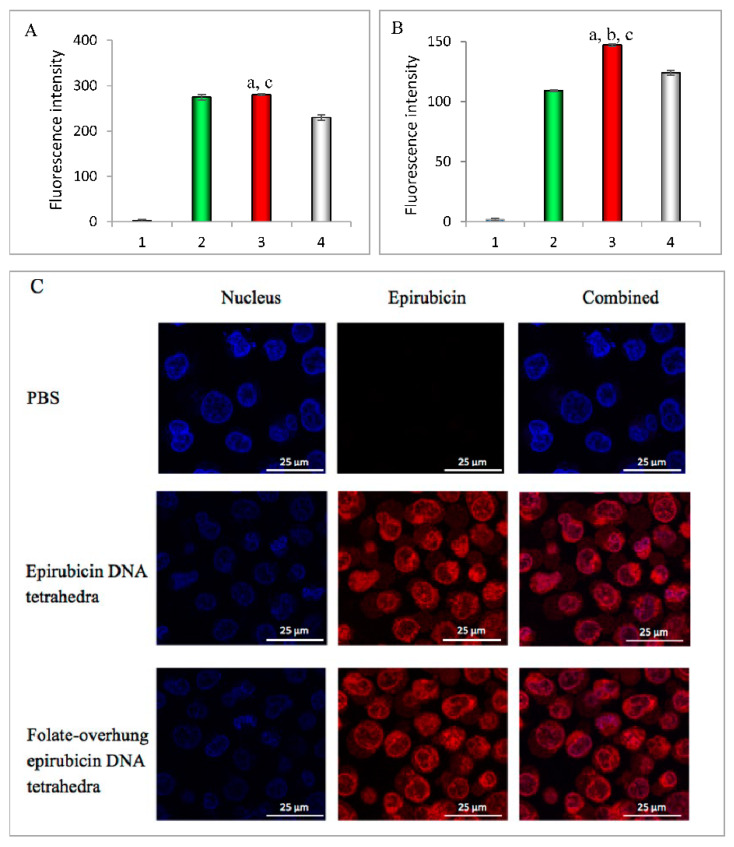
Targeted capture of folate-overhung DNA tetrahedra by leukemic cells and their co-localization into nuclei, by transport across the nuclei membrane. Epirubicin was used as a fluorescent probe for indicating folate-overhung epirubicin DNA tetrahedra. (**A**). Cellular uptake by BALL-1 cells indicated with the fluorescence intensity; (**B**). Cellular uptake by K562 cells indicated with the fluorescence intensity. For (**A**,**B**): 1. PBS; 2. Epirubicin DNA tetrahedra; 3. Folate-overhung epirubicin DNA tetrahedra; 4. Folate-overhung epirubicin DNA tetrahedra after saturation with excess of folate. a. vs. PBS; b. vs. epirubicin DNA tetrahedra; c. vs. folate-overhung epirubicin DNA tetrahedra after folate saturation. *p* < 0.05 (**C**). The co-localization of drug with nuclei of leukemia K562 cells.

To display the targeted capture of folate-overhung DNA tetrahedra by cancer cells, the cellular uptakes were performed on leukemia BALL-1 and K562 cells. To indicate this, epirubicin, a structural analog of mitoxantrone, was used as a fluorescent probe. When applying drug formulations to the BALL-1 cells, the cellular uptake ranking was folate-overhung epirubicin DNA tetrahedra > epirubicin DNA tetrahedra in BALL-1 cells (Figure 2A). When applying drug formulations to the K562 cells, the cellular uptake ranking was folate-overhung epirubicin DNA tetrahedra > epirubicin DNA tetrahedra (Figure 2B).

To prove these outcomes, folate was pre-added to block the folate receptor in both cells, and the results showed that the cellular uptakes of folate-overhung epirubicin DNA tetrahedra were decreased.

### 3.3. Transporting across the Nuclei Membrane

To indicate the transport capability of the folate-overhung DNA tetrahedra across the nuclei membrane, a confocal microscope was utilized to evaluate the co-localization of different formulations with the nuclei of leukemic cells. Similarly, epirubicin was used as a fluorescent probe. In the images, the red fluorescence represented epirubicin, while the blue fluorescence indicated the nuclei. The results showed that epirubicin, epirubicin DNA tetrahedra, and folate-overhung epirubicin DNA tetrahedra could be co-localized with the nuclei of K562 cells (Figure 2C) and BALL-1 cells (Figure S2).

### 3.4. Enhancing the Killing Effect in Leukemic Cells

**Figure 3:**
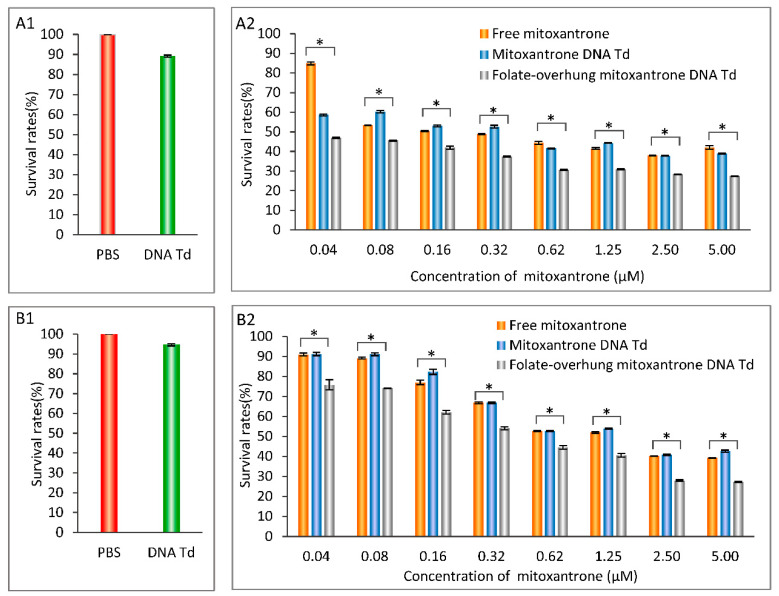
Cytotoxicity of folate-overhung mitoxanthrone DNA tetrahedra in leukemia BALL-1 and K562 cells (**A1**). Survival rates (%) of the BALL-1 cells after the treatment with varying formulations (PBS, DNA tetrahedra) at 48 h; (**A2**). Survival rates (%) of the BALL-1 cells after the treatment with varying formulations (free mitoxantrone, mitoxantrone DNA tetrahedra, folate-overhung mitoxantrone DNA tetrahedra) at 48 h; (**B1**). Survival rates (%) of the K562 cells after the treatment with varying formulations (PBS, DNA tetrahedra) at 48 h; (**B2**). Survival rates (%) of the K562 cells after treatment with varying formulations (free mitoxantrone, mitoxantrone DNA tetrahedra, folate-overhung mitoxantrone DNA tetrahedra) at 48 h.* signifies *p* < 0.05.

To demonstrate the targeted anti-cancer efficacy of folate-overhung DNA tetrahedra in vitro, cytotoxic effects were evaluated on leukemia BALL-1 cells and K562 cells. The results showed that the ranking of anti-cancer efficacy was folate-overhung mitoxantrone DNA tetrahedra > mitoxantrone DNA tetrahedra, in both the BALL-1 cells (Figure 3(A1, A2)) and the K562 cells (Figure 3(B1,B2)).

### 3.5. Initiating Apoptosis of Leukemic Cells

**Figure 4:**
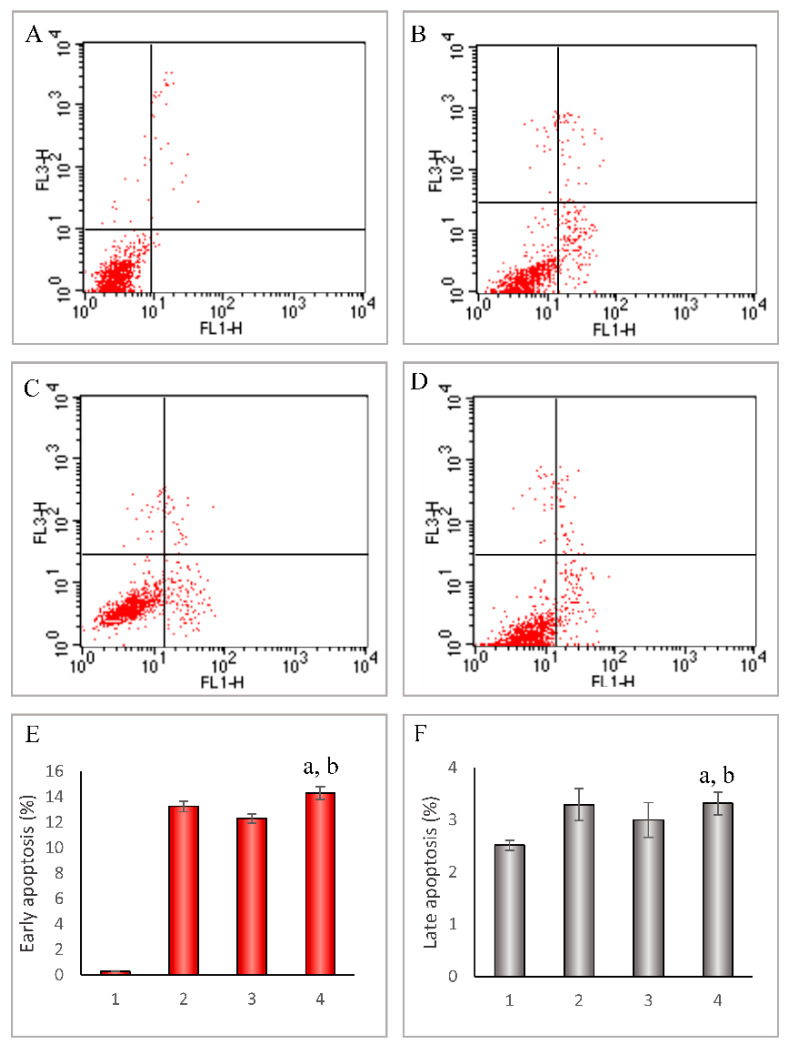
Induced apoptosis in leukemia BALL-1 cells by folate-overhung mitoxantrone DNA tetrahedra. (**A–D**): Induced apoptosis after treatment with varying formulations at 10 h, and the cells were stained using Annexin V-Fluor 647 and 7AAD, then assessed according to manufacturer instructions by the FACScan flow cytometer. UR: Late apoptosis and dead cells; LR: Early apoptosis cells (**A**). PBS; (**B**). Free mitoxantrone; (**C**). mitoxantrone DNA tetrahedra; (**D**). Folate-overhung mitoxantrone DNA tetrahedra; (**E**). The early apoptosis rate in BALL-1 cells (**F**). The late apoptosis rate in BALL-1 cells. (**E–F**): 1. PBS; 2. Free mitoxantrone; 3. Mitoxantrone DNA tetrahedra; 4. Folate-overhung mitoxantrone DNA tetrahedra. a. vs. PBS; b. vs. mitoxantrone DNA tetrahedra, *p* < 0.05.

To indicate the increased apoptosis by folate-overhung mitoxantrone DNA tetrahedra in leukemia, the apoptosis rates and the mechanism were studied in the BALL-1 and K562 cells. After applying two kinds of mitoxantrone-containing DNA tetrahedra, the apoptosis rate ranking was folate-overhung mitoxantrone DNA tetrahedra > mitoxantrone DNA tetrahedra, in both BALL-1 cells and K562 cells (Figure 4 and Figure S3).

**Figure 5:**
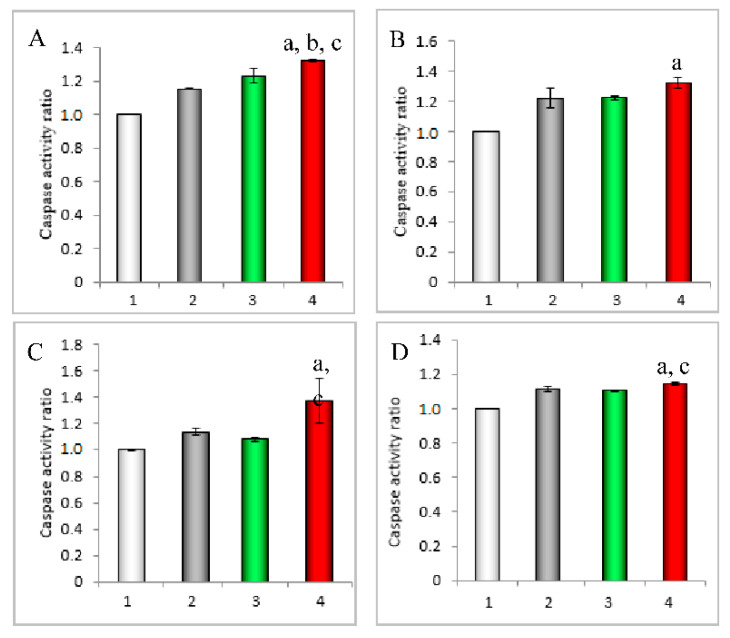
Activated caspase-8 and caspase-3 in leukemia BALL-1 cells and K562 cells by folate-overhung mitoxantrone DNA tetrahedra at 12 h (**A**). Caspase-8 activity ratio in BALL-1 cells; (**B**). Caspase-3 activity ratio in BALL-1 cells; (**C**). Caspase-8 activity ratio in K562 cells; (**D**). Caspase-3 activity ratio in K562 cells. For (**A–D)**: 1. Blank; 2. PBS; 3. Mitoxantrone DNA tetrahedra; 4. Folate-overhung mitoxantrone DNA tetrahedra. a. vs. blank; b. vs. PBS; c. vs. mitoxantrone DNA tetrahedra, *p* < 0.05.

To further reveal the action mechanism, the activities of upstream apoptotic enzyme caspase-8, and downstream apoptotic enzyme caspase-3 were measured in the BALL-1 cells and K562 cells. In two kinds of leukemic cells, folate-overhung mitoxantrone DNA tetrahedra showed an increased caspase-8 activity (Figure 5A,C), and an increased caspase-3 activity (Figure 5B,D), as compared to mitoxantrone DNA tetrahedra, as compared to the control formulations.

### 3.6. Enhancing the Efficacy in Leukemia Xenografts in Mice

**Figure 6:**
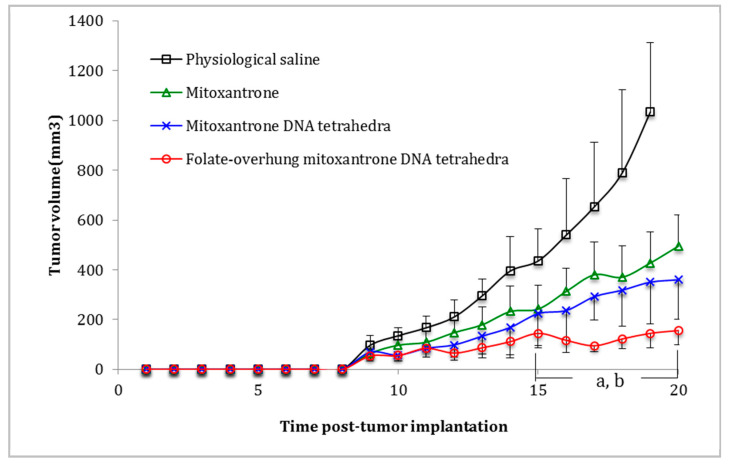
Anticancer efficacy in leukemia BALL-1 cell xenografts in nude mice after treatment with Figure 1. tumor volumes after treatment with different formulations in nude mice; a. vs. mitoxantrone, b. vs. mitoxantrone DNA tetrahedra, *p* < 0.1.

To verify the anticancer efficacy of folate-overhung mitoxantrone DNA tetrahedra in vivo, leukemia BALL-1 cells were xenografted into the nude mice. After drug administration, the results displayed that the ranking on tumor volume inhibition was folate-overhung mitoxantrone DNA tetrahedra > mitoxantrone DNA tetrahedra > free mitoxantrone > physiological saline (Figure 6). Furthermore, the survival curves demonstrated that all mice treated with folate-overhung mitoxantrone DNA tetrahedra were still alive during the whole experiment. In contrast, those treated with physiological saline were dead, with a mortality of 100% (Figure S4). Moreover, the analyses on the body weight showed that there were no significant differences between varying drug formulations (Figure S5). Nevertheless, the administration of mitoxantrone DNA tetrahedra or folate-overhung mitoxantrone DNA tetrahedra caused a mild increase in white blood cells, a decrease in red blood cells, and a decrease in hemoglobin (Table S3).

## 4. Discussion

In this study, we construct a precise nanostructure of folate-overhung mitoxantrone DNA tetrahedron based on the Watson–Crick base complementary pairing principle. DNA tetrahedron was used as a drug carrier because of its unique natures, consisting of precisely controllable, self-assemble, biocompatible, and targetable properties. Due to steric hindrance, DNA tetrahedron has the ability to resist nuclease attack and maintains its structural integrity over the course of a long period. As compared to a single nucleic acid, DNA tetrahedron can independently penetrate negatively charged cell membranes without any ligand or transfection agent and then deliver the drug into cancer cells [15,16].

To further increase the targetable property of DNA tetrahedron, folic acid is used by synthesizing a folate-overhung DNA tetrahedron, which is able to enrich the tetrahedron onto cancer cells due to the overexpression of folate receptors on the cell membrane surface in many cancer cells, including leukemic cells [17]. Actually, based on current knowledge, there are less specific receptors expressed on cancer cells, because receptors usually express in both normal cells and cancer cells. Nevertheless, cancer cells often overly express some specific receptors, like folate receptor, and this would allow a remarkable targeted efficiency by folate ligand modified DNA tetrahedron nanostructure, thereby potentially reducing the systemic toxicity of mitoxanthrone carried.

It has been found that at least three folate molecules are needed for each nanoparticle to achieve optimal drug delivery [18]. Therefore, we chose to synthesize six folate molecules on each overhung DNA tetrahedron. The results demonstrate a successful synthesis of such a folate-overhung mitoxantron DNA tetrahedron (Figure 1A–D and Figure S1).

Furthermore, the results reveal that the folate-overhung mitoxantrone DNA tetrahedron has appropriate particle size, high encapsulation efficiency, slow drug leaky effect and suitable stability (Figure 1E–G, and Table S2). The nano-size (25 nm) enables the optimal particle size to transfer the carrier across the vasculature in tumor tissue at a range of 20–200 nm [19,20,21], hence extending its application of folate-overhung mitoxantrone DNA tetrahedron to treat the solid tumor. The slower drug leaky effect and suitable stability (Figure 1H) could assign the possibilities of this formulation a stable characteristics in the blood system, not being degraded by enzyme system, and less leaking before reaching the leukemia cells. The tetrahedron can be formed by incubation at room temperature as well (data not shown), but the yield is reduced [22], as compared to that of PCR engineering.

To evaluate the targeting effect of the folate-overhung DNA tetrahedron on BALL-1 and K562 cells in vitro, epirubicin is used as a fluorescent probe, as both mitoxantrone and epirubicin belong to anthracycline structure, that can be easily intercalated into DNA tetrahedron, while mitoxanthrone does not have fluorescence. The results demonstrate that the folate-overhung epirubicin DNA tetrahedron can be mostly captured by leukemic cells, and further delivered into the nuclei. These suggest the targeted capability of the structure, and would be beneficial for mitoxantrone to take effect in the nuclei of leukemic cells (Figure 2 and Figure S2). In addition, folate unmodified DNA tetrahedron also demonstrates a strong capture by leukemic cells. It may be explained by the fact that, unlike epirubicin and mitoxanthrone, both of which are the substrate of ATP-binding cassette (ABC) transporters (efflux pumps of exogenous molecules), DNA tetrahedron can avoid the recognition of efflux pump, prevent the degradation of endosomes in the cytoplasm [23,24], and easily enter into the nuclei. The cellular uptake of folate-overhung DNA tetrahedron also exhibits differences in two kinds of leukemic cells. This may be due to the difference of the expressed folate receptors in the K562 cells and in the BALL-1 cells.

Unlike solid tumor, most of the leukemic cells may be distributed in the whole body, especially in the circulation system. This increases the difficulty in the regular chemotherapy. Therefore, the targeted chemotherapy can provide a beneficial opportunity to eliminate the leukemic cells. The experimental results demonstrate that the folate-overhung mitoxantrone DNA tetrahedra have the strongest cytotoxicity in the leukemic cells, as compared with the control formulations. They indicate a strong cytotoxicity to leukemia cells, in particular, at a low concentration (<1 μM mitoxantrone) (Figure 3). The data also reveal that there exists different toxicities towards these two kinds of leukemic cells. The difference in the survival rates of BALL-1 and K562 may be due to the difference in the drug response to mitoxantrone and drug resistant nature of the cells.

The total efficacy of an anticancer drug is achieved by directly killing cancer cells and inducing apoptosis. Accordingly, the targeted chemotherapy-induced apoptosis could be used as an effective approach for increasing the efficacy during treatment. In addition to the direct killing effect, the results demonstrate that the targeted folate-overhung mitoxantrone DNA tetrahedron induces evident apoptosis in two kinds of leukemic cells (Figure 4 and Figure S3). The mechanism for the induced apoptosis is associated with the activated signaling pathway from initiator caspase-8 to effector caspase-3 [25], thereby initiating a cascade of apoptotic reactions in leukemic cells (Figure 5).

To confirm the in vivo efficacy, a mouse model of leukemic cell xenografts was successfully established. The results demonstrate that the folate-overhung mitoxantrone DNA tetrahedra can significantly inhibit the growth of the xenografted tumors, as compared to mitoxantrone DNA tetrahedra or free mitoxantrone (Figure 6). In addition, the survivals of mice are also extended by treating with folate-overhung mitoxantrone DNA tetrahedra or by treating with mitoxantrone DNA tetrahedra as well, as compared to that with free mitoxantrone (Figure S4). These results are consistent with the results of in vitro studies. The body weight changes and blood indicators of nude mice are used as preliminary safety indicators to evaluate the safety of the formulations (Figure S5, and Table S3). The results indicate that the folate-overhung mitoxantrone DNA tetrahedra significantly enhances the survival of the leukemia bearing animals with a safe property. It is worth noting that, in viewing tumor inhibition efficacy (Figure 6), survival (Figure S4), body weight (Figure S5) and blood indicator (Table S3) of the treated mice, the non-targeted mitoxantrone DNA tetrahedra demonstrate a promising anticancer efficacy, and safety as well. This may suggest that DNA tetrahedra also show the potential as a kind of drug carrier for delivering drug into the nuclei of cancer cells [23,24]. Nevertheless, folate-overhung mitoxantrone DNA tetrahedra exhibit more favorable features due to their targeted property. In addition, as most of the leukemic cells may be distributed in the whole body, a non-solid tumor leukemia mouse model may need to be established in further study.

## 5. Conclusions

We precisely synthesized a folate-overhung mitoxantrone DNA tetrahedron (approximately 25nm) by incorporating six folate molecules, which were separately synthesized on 6 DNA overhang complements, and by intercalating mitoxantrone into DNA double helix strands. The folate-overhung mitoxantrone DNA tetrahedra were able to targeted capture leukemic cells, transport across the nuclei membrane, induce the apoptosis, and enhance the overall efficacy in treating leukemic cells in vitro and in leukemia-bearing mice. The study indicates that the folate-overhung DNA tetrahedron offers a promising drug-containing DNA nanostructure to clean the sparsely distributed leukemic cells in patients.

## Supplementary Materials

The following are available online at https://www.mdpi.com/2079-4991/10/5/951/s1, Figure S1: Synthesis of the folate-overhang complement; Figure S2: Co-localization with nuclei in leukemia BALL-1 cells after treatment with varying formulations at 2 h; Figure S3: Induced apoptosis in leukemia K562 cells after treatment with folate-overhung mitoxantrone DNA tetrahedra; Figure S4: Anticancer efficacy in leukemia BALL-1 cell xenografts in nude mice after treatment with folate-overhung mitoxantrone DNA tetrahedra. Survival number of nude mice after treatment with different formulations; Figure S5: Body weight of nude mice after tumor implantation; Table S1: Base sequences of strands for synthesis of DNA tetrahedra; Table S2: Characterization of the DNA tetrahedra; Table S3: Blood indicators of nude mice after treatment with varying formulations.
