# Supplementary material for: A Precise Nanostructure of Folate-Overhung Mitoxantrone DNA Tetrahedron for Targeted Capture Leukemia
Source: Nanomaterials (Basel). 2020 May 16;10(5):951. doi: 10.3390/nano10050951 (PMC7279534; doi:10.3390/nano10050951)
Supplement: Supplementary file 1 [file nanomaterials-10-00951-s001.pdf]

Supporting Information

A Precise Nanostructure of Folate-Overhung Mitoxantrone DNA Tetrahedron for Targeted CAPTURE leukemia

Ying-Zi Bu, Jia-Rui Xu, Qian Luo, Ming Chen, Li-Min Mu and Wan-Liang Lu *

State Key Laboratory of Natural and Biomimetic Drugs, Beijing Key Laboratory of Molecular Pharmaceutics and New Drug System, School of Pharmaceutical Sciences, Peking University, Beijing 100191, China; eiko@pku.edu.cn (Y.-Z.B.); xujr@bjmu.edu.cn (J.-R.X.); lq-ql@pku.edu.cn (Q.L.); cm_cally@pku.edu.cn (M.C.); liminmu@bjmu.edu.cn (L.-M.M.)

* Correspondence: luwl@bjmu.edu.cn; Tel.: +86-10 82802683

Received: 10 March 2020; Accepted: 14 May 2020; Published: 16 May 2020

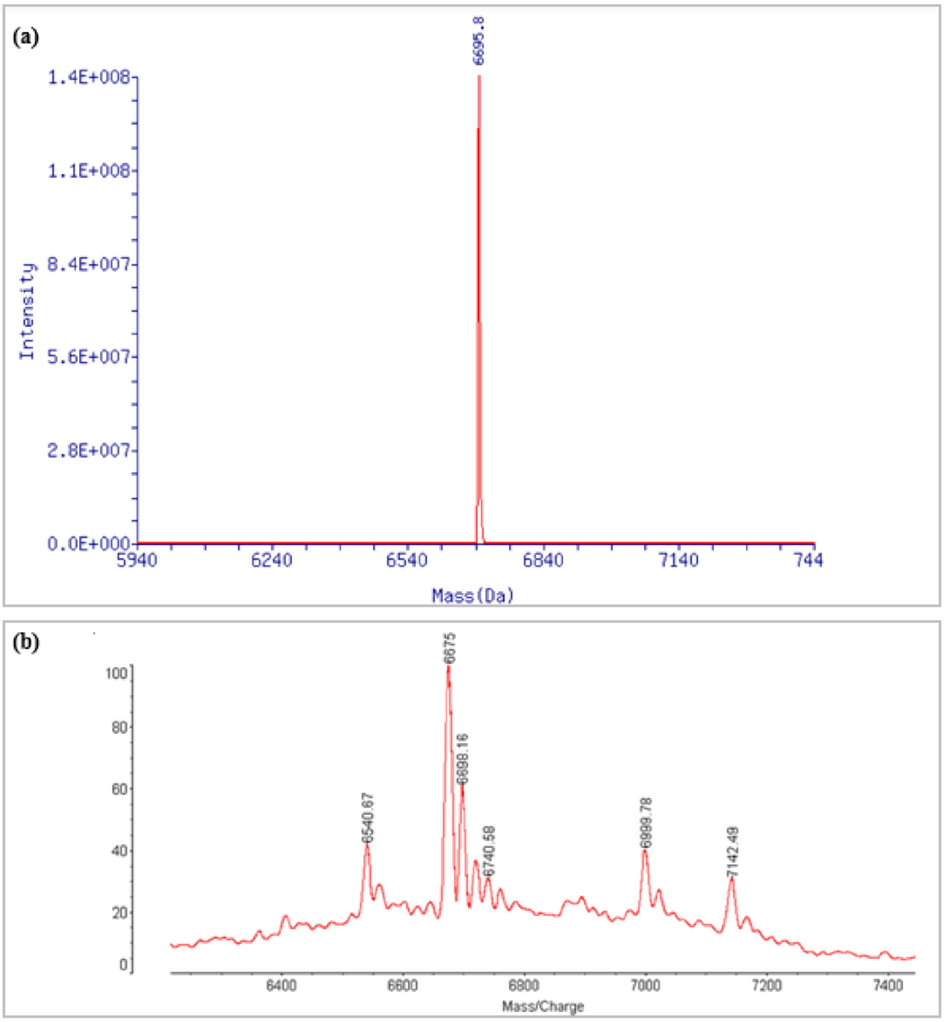

Figure S1. Synthesis of the folate-overhang complement. (**a**) MALDI-TOF-MS spectrum of the overhang complement; (**b**) MALDI-TOF-MS spectrum of the synthesized folate-overhung complement.

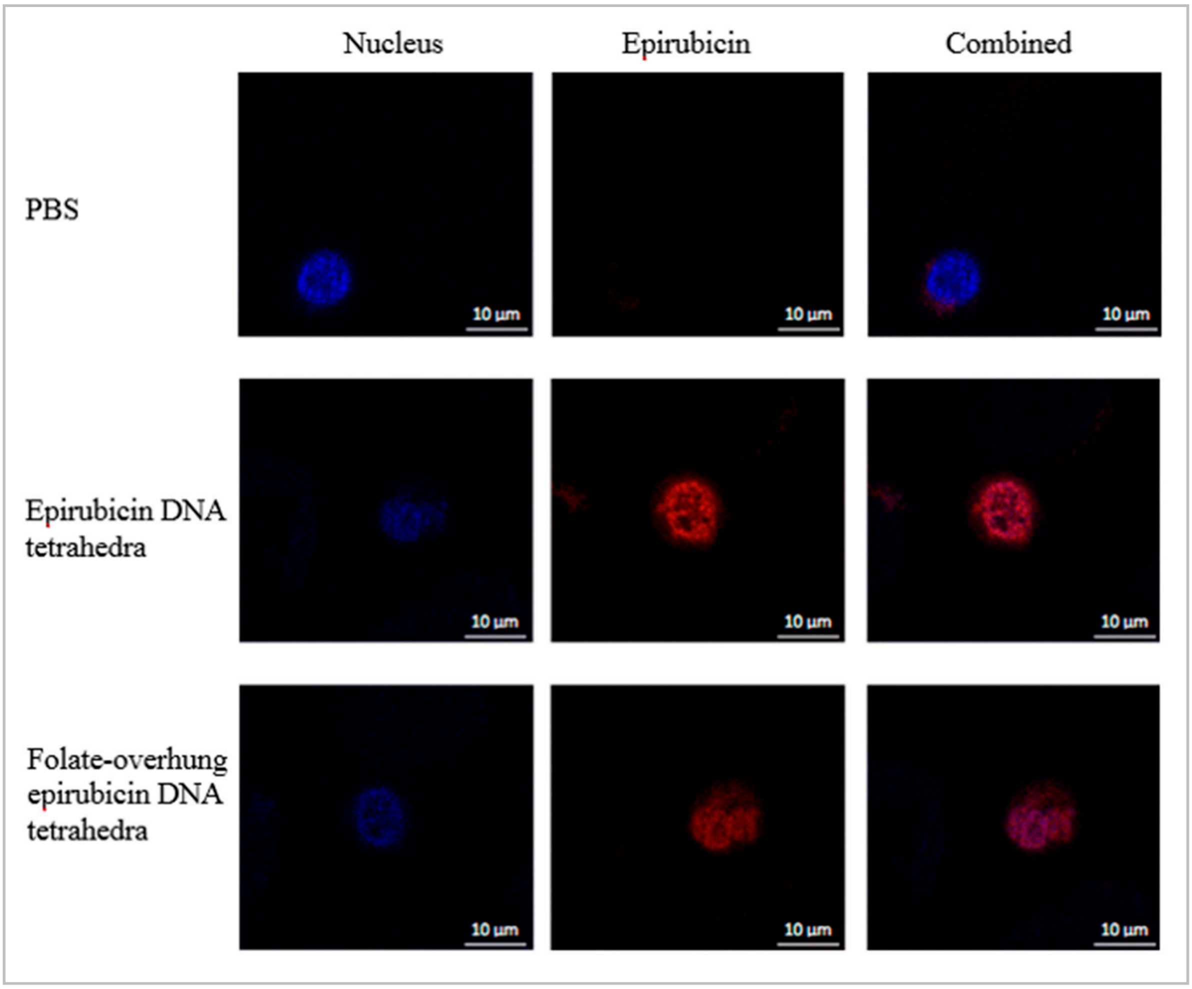

Figure S2. Co-localization with nuclei in leukemia BALL-1 cells after treatment with varying formulations at 2 h.

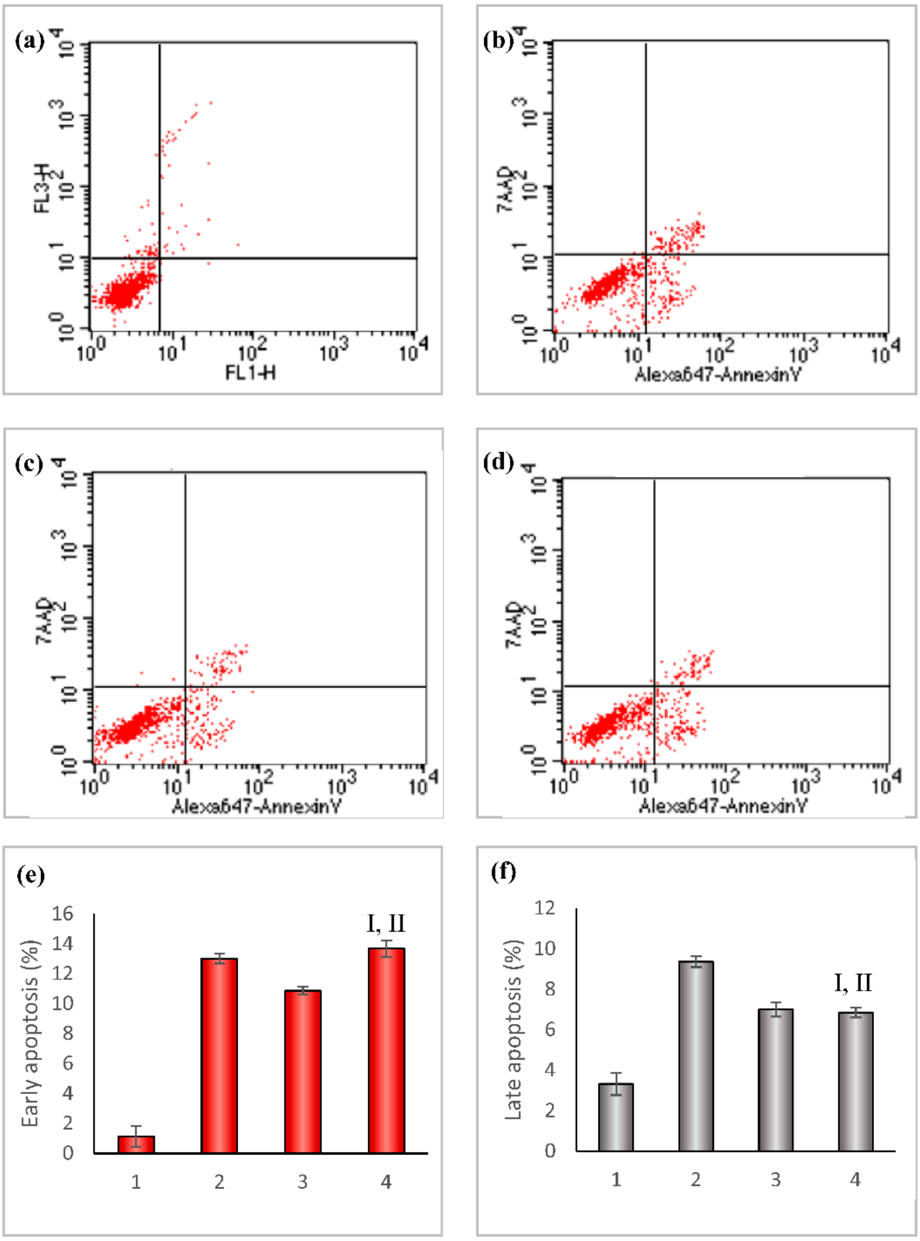

Figure S3. Induced apoptosis in leukemia K562 cells after treatment with folate-overhung mitoxantrone DNA tetrahedra. (**a**–**d**): Induced apoptosis after treatment with varying formulations at 10 h, and the cells were stained using Annexin V-Fluor 647 and 7AAD, then assessed according to manufacturer instructions by the FACScan flow cytometer. UR: Late apoptosis and dead cells; LR: Early apoptosis cells. (**a**) PBS; (**b**) Free mitoxantrone; (**c**) Mitoxantrone DNA tetrahedra; (**d**) Folate-overhung mitoxantrone DNA tetrahedra. (**e**) The early apoptosis rate in K562 cells. (**f**) The late apoptosis rate in K562 cells. For (**e**)-(**f**): 1. PBS; 2. Free mitoxantrone; 3. Mitoxantrone DNA tetrahedra; 4. Folate-overhung mitoxantrone DNA tetrahedra. I. vs PBS; II. vs mitoxantrone DNA tetrahedra, $p < 0.05$.

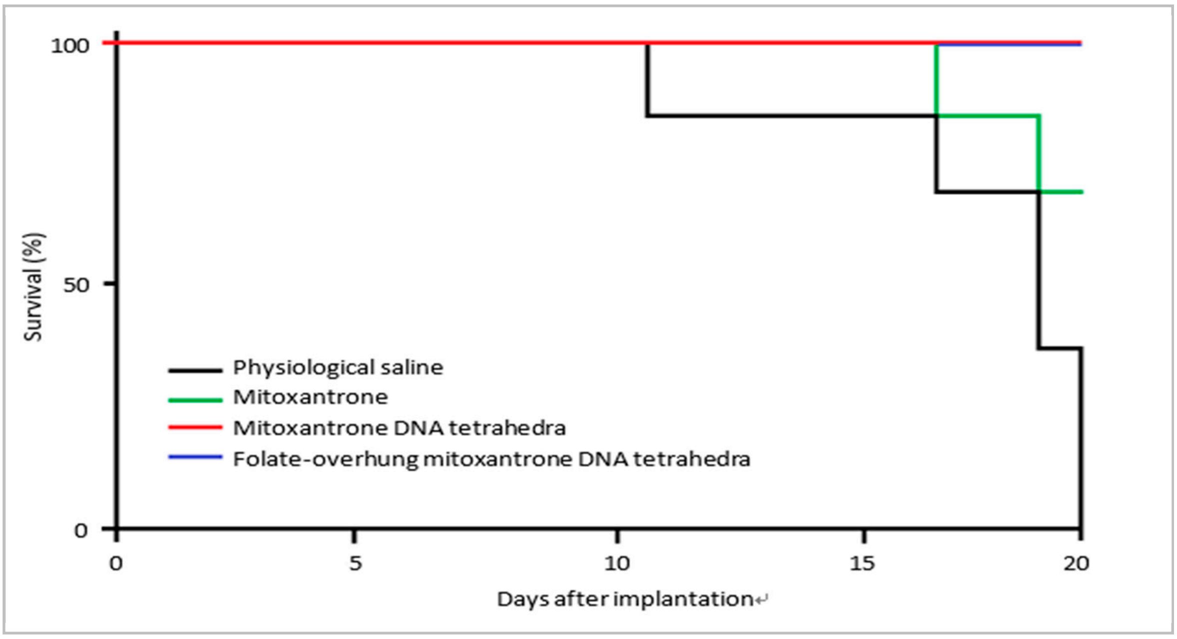

Figure S4. Anticancer efficacy evaluated by survival curve in leukemia BALL-1 cell xenografts in nude mice after treatment with folate-overhung mitoxantrone DNA tetrahedra. .

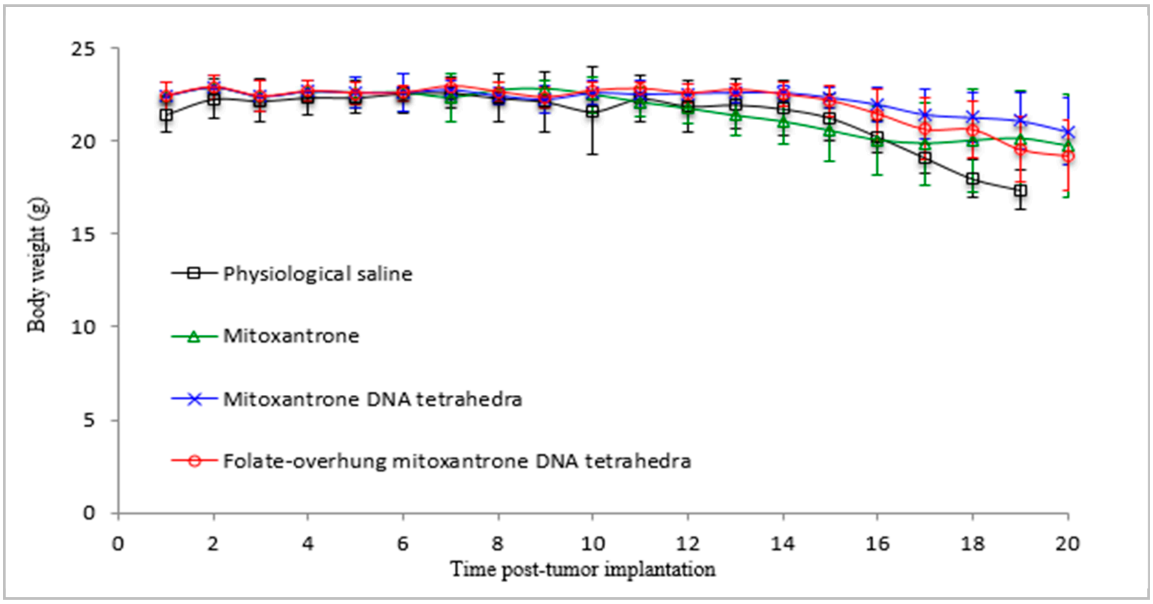

Figure S5. Body weight of nude mice after tumor implantation.

Table S1. Base sequences of strands for synthesis of DNA tetrahedra.

DNA strands	DNA sequences (5' -3')
Strand 1	GTCTGAGGCAGTTGAGAGATCTCGAACATTCC
Strand 2	TAAGTCTGAAGATCCATTTATCACCAGCTGCTGCACGCCATAGT AGACGTATCACCTGTCC
Strand 3	AGCTACTTGCTACACGAGGATCTTCAGACTTAGGAATGTTCGAG ATCACATGCGAGGACTCGGTCCAATACCGTACTAACGATTACAG ATCAA
Strand 4	CAGCTGGTGATAAAACGTGTAGCAAGTAGCTTTGATCTGTAATC GACTCTACGGGAAGAGC
Strand 5	ATGCCCATCCGGCTCACTACTATGGCGTGCAG
Strand 6	CGAGTCCTCGCATGACTCAACTGCCTCAGACGGACAGGTGATA CGAGAGCCGGATGGGCATGCTCTTCCCGTAGAGACGGTATTGGA CATGAT
Overhang strand	TTTTTTTTTTTTTTTTTTTT
Overhang complement	AAAAAAAAAAAAAAAAAAAA

Table S2. Characterization of the DNA tetrahedra.

	Mitoxantrone DNA tetrahedra	Folate-overhung mitoxantrone DNA tetrahedra
Encapsulation efficiency (%)	86.16 ± 0.34	94.73 ± 0.31
Content (mg/ml)	0.96 ± 0.002	1.04 ± 0.007
Leaky rate (%) at 24 h	6.12 ± 0.95	6.45 ± 0.66

Notes: Data are presented as mean ± standard deviation (n = 3).

Table S3. Blood indicators of nude mice after treatment with varying formulations.

	Blank	Physiological saline	Mitoxantrone	Mitoxantrone DNA tetrahedra	Folate-overhung mitoxantrone DNA tetrahedra
WBC	8.3 ± 2.60	9.2 ± 1.73	9.3 ± 1.04	11.3 ± 3.02	11.2 ± 6.38
RBC	10.20 ± 0.53	9.04 ± 0.50	8.77 ± 0.51	8.64 ± 0.60	8.96 ± 0.64
HGB	146 ± 6.34	135 ± 8.70	137 ± 7.92	131 ± 7.31	137 ± 6.67
HCT	43.4 ± 2.28	39.0 ± 2.42	38.2 ± 1.86	37.3 ± 2.44	38.0 ± 2.19
MCV	42.5 ± 1.19	43.2 ± 0.76	43.7 ± 1.40	43.2 ± 0.84	42.4 ± 0.99
MCH	14.4 ± 0.43	15.0 ± 0.50	15.7 ± 0.76	15.2 ± 0.27	15.3 ± 0.54
MCHC	337 ± 3.03	346 ± 5.41	359 ± 5.89	352 ± 4.72	361 ± 7.23[I, II]
RDW	13.9 ± 0.31	14.0 ± 0.57	13.7 ± 0.27	14.1 ± 0.60	13.8 ± 0.26
PLT	794 ± 64.49	835 ± 81.34	861 ± 37.74	781 ± 112.56	696 ± 126.68
PCT	0.30 ± 0.03	0.32 ± 0.03	0.32 ± 0.02	0.31 ± 0.04	0.25 ± 0.05
MPV	3.8 ± 0.11	3.8 ± 0.13	3.8 ± 0.09	4.0 ± 0.17	3.7 ± 0.23
PDW	13.2 ± 0.42	13.9 ± 0.54	13.8 ± 0.53	13.6 ± 0.55	13.5 ± 0.65
LYM	1.2 ± 0.37	1.0 ± 0.44	0.75 ± 0.23	1.1 ± 0.37	1.2 ± 0.56
MID	0.6 ± 0.18	0.5 ± 0.33	0.2 ± 0.17	0.5 ± 0.38	0.3 ± 0.13
GRN	6.5 ± 2.49	7.6 ± 1.53	8.3 ± 1.32	9.8 ± 3.18	9.7 ± 6.23
LYM	15.7 ± 5.00	11.5 ± 3.48	8.35 ± 3.42	11.2 ± 6.54	14.6 ± 13.30
MID	7.1 ± 1.99	5.6 ± 3.15	2.9 ± 2.20	4.18 ± 3.31	3.08 ± 1.81
GRN (%)	77.2 ± 6.87	82.9 ± 6.02	88.8 ± 5.35	84.6 ± 8.54	82.3 ± 14.71

Notes: Data are presented as mean ± standard deviation (n = 4). I. *vs* blank, II. *vs* mitoxantrone, $p < 0.05$.
